# Association between thyroid hormone levels in the acute stage of stroke and risk of poststroke depression: A meta‐analysis

**DOI:** 10.1002/brb3.3322

**Published:** 2023-12-26

**Authors:** Jie Fu, Qin Zhao, Jinglun Li, Xiu Chen, Lilei Peng

**Affiliations:** ^1^ Department of Neurology The Affiliated Hospital of Southwest Medical University Luzhou Sichuan China; ^2^ Department of Neurosurgery The Affiliated Hospital of Southwest Medical University Luzhou Sichuan China

**Keywords:** depression, meta‐analysis, poststroke depression, stroke, thyroid hormones

## Abstract

**Background:**

Thyroid hormones have been indicated to be associated with depression, but their relationship with poststroke depression (PSD) remains controversial. Therefore, we performed a meta‐analysis to explore the correlation between thyroid hormone levels in acute stroke and PSD.

**Methods:**

We searched databases for eligible studies. Standard mean differences (SMD) and 95% confidence intervals (CI) were applied to evaluate the association among levels of thyroid hormones, including thyroid‐stimulating hormone (TSH), free triiodothyronine (FT3), and free thyroxine (FT4), in acute stroke patients and the risk of PSD.

**Results:**

A total of 13 studies were included in the analysis. Compared to non‐PSD patients, PSD patients had remarkably lower serum TSH and FT3 levels (TSH: SMD = −0.59, 95%CI = −1.04 to −.15, *p* = .009; FT3: SMD = −0.40, 95%CI = −.51 to −.30, *p* = .000) and higher serum FT4 levels (SMD = 0.33, 95%CI = .07–.59, *p* = .013). Subgroup analysis showed that there may be a more statistically significant association between FT3 and the risk of PSD compared to TSH and FT4.

**Conclusions:**

Our results suggested that patients with lower serum TSH and FT3 levels as well as higher serum FT4 levels in the acute stage of stroke may be more susceptible to PSD.

## Graphical abstract

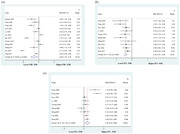

## 1 INTRODUCTION

Poststroke depression (PSD) is one of the frequent neuropsychiatric consequences after stroke (Levada & Troyan, 2018). Approximately one third of stroke survivors suffered from PSD (Guo, Wang, Sun, et al., 2022). The frequency of PSD is highest during the first month after stroke and remains high even after several years (Göthe et al., 2012). Risk factors of PSD are diverse, including stroke location and severity, recurrent stroke, poor social support, as well as personal factors, such as age, gender, and history of psychiatric disorders (Medeiros et al., 2020). The pathogenesis of PSD is complex, and several hypotheses have been proposed, including monoamine neurotransmitter dysregulation (Hama et al., 2017), inflammatory response (Yang et al., 2022), hypothalamic–pituitary–adrenal axis hypothesis (Zhou et al., 2022), and hypothalamus–pituitary–thyroid axis imbalance (Taroza et al., 2019). PSD hinders the process of stroke rehabilitation and is associated with poor functional outcomes and an increased risk of all‐cause mortality (Cai et al., 2019; Nickel & Thomalla, 2017). Therefore, early detection of PSD in stroke patients and recognizing people at risk of developing PSD are of importance, which could lead to the implementation of early intervention.

Thyroid hormones are important for brain development and play an essential role in maintaining brain functions throughout life (Talhada et al., 2019). The thyroid gland generates and releases predominantly the prohormone thyroxine (T4) along with a small quantity of the bioactive hormone triiodothyronine (T3), which is regulated by thyroid‐stimulating hormone (TSH) secreted by the pituitary gland. In blood circulation, a portion of the T4 is converted to T3 via a process called deiodination. Both T4 and T3 have a bound form and a free form in the peripheral blood. The free form is also an active form, which enters relevant body tissues and exerts biological functions, whereas the bound form attaches to proteins, which makes it biologically inert (Jiang et al., 2017). Stroke could cause changes in thyroid function in nonthyroidal illness, at least in the acute phase (Theodoropoulou et al., 2006). A cross‐sectional study revealed that the prevalence of thyroid hormone alterations among acute ischemic stroke patients without known thyroid diseases was 17.8% (13/73) (Bashyal et al., 2021). Low T3 syndrome is characterized by low free T3 (FT3) level together with normal‐to‐low free T4 (FT4) and TSH levels (Gao et al., 2018), and it is reported that low T3 syndrome affects 32%–62% of patients undergoing acute cerebrovascular diseases (Bunevicius et al., 2015). Moreover, a recent study investigated dynamics of thyroid function after acute ischemic stroke, and they found that thyroid hormones, particularly FT3, declined shortly after the beginning of stroke, bottomed during the first days, and then recovered in the chronic stage (Sidorov et al., 2023). Additionally, thyroid abnormalities are indicated to be related to the occurrence and development of depression (Berent et al., 2014; Duntas & Maillis, 2013). Even variations in thyroid hormones within the normal range could also be risk factors for depression (Medici et al., 2014). These evidences revealed the potential relationship among thyroid hormone changes in acute stroke patients without thyroid diseases and the risk of PSD. However, there were inconsistent results in the comparisons of thyroid hormone levels in the acute stage of stroke between stroke patients with PSD and without PSD (non‐PSD) based on previous studies. Taroza et al. (2019) reported that serum FT3 levels were remarkably lower in acute stroke patients with PSD compared to non‐PSD stroke survivors, whereas the study from Guo, Wang, Sun, et al. (2022) and Guo, Wang, Xia, et al. (2022) showed no significant difference in serum FT3 levels between the two groups. Therefore, we systematically reviewed the evidence and performed a meta‐analysis to validate the hypothesis that thyroid hormone levels in the acute stage of stroke might be linked to PSD susceptibility. Given that the thyroid hormones investigated in most of the current studies were mainly TSH, FT3, and FT4, our meta‐analysis also focused on these three indices.

## 2 METHODS

Results of our study were reported according to the recommendations of the Preferred Reporting Items for Systematic Reviews and Meta‐Analyses (PRISMA) statement (Moher et al., 2009). Ethical approval and patient consent were not required because our study was retrieved based on previous published studies.

### 2.1 Literature search

PubMed, Cochrane Library, Web of Science, Weipu, Wanfang, and China National Knowledge Infrastructure (CNKI) databases were searched up to February 2023. The search terms used were as follows: (“post‐stroke depression” OR “poststroke depression” OR “depression after stroke” OR “PSD”) AND (“thyroid function” OR “thyroid hormones” OR “thyroid‐stimulating hormone” OR “free triiodothyronine” OR “free thyroxine” OR “TSH” OR “FT3” OR “FT4”). Previous reviews were also searched to identify additional trials. There was no language restriction.

### 2.2 Trial selection

Studies were included if they met the following criteria: (1) observational studies; (2) patients were diagnosed as transient ischemic attack (TIA), acute ischemic or hemorrhagic stroke; (3) patients had no previous history of depression and thyroid diseases; (4) blood samples to test serum TSH, FT3, and FT4 levels were collected in the acute stage of stroke; (5) comparative data on serum TSH, FT3, and FT4 levels between PSD and non‐PSD could be extracted. The exclusion criteria were as follows: (1) repetitive data; (2) conference abstracts, case reports, letters, reviews, and meta‐analysis. Two investigators (ZQ and LJL) independently evaluated the eligibility of literature, and any disagreements were resolved by a discussion with a third investigator (FJ).

### 2.3 Data extraction

Two researchers (FJ and ZQ) independently extracted the following data from each of the included studies: author, publication year, country, types of stroke, methods of diagnosis of stroke, and PSD, time points when PSD was evaluated, sample size, mean age (years), sex, serum thyroid hormone levels (TSH, FT3, and FT4), and study design. The data on serum thyroid hormone levels were reported as medians and interquartile ranges, which were converted to the form of mean and standard deviation (SD) by using the Box–Cox method (McGrath et al., 2020). Any discrepant data were negotiated by the investigators to ensure accurate data were obtained.

### 2.4 Evaluation of quality of studies

The Newcastle–Ottawa scale (NOS) criteria were used to evaluate the study quality. The NOS evaluates the quality of the study according to three categories, including selection, comparability, and outcome (cohort studies) or exposure (case–control studies) (Wells et al., 2009). The total score ranged from 0 to 9, and a study was regarded as high quality when the total score is not less than 5.

### 2.5 Statistical analysis

The meta‐analysis was performed by using the STATA 17.0. A *p* value with <.05 in *Z* test was regarded statistically significant. All outcomes in this meta‐analysis were continuous data, so data were presented as standard mean difference (SMD) and 95% confidence intervals (CI). The total SMD was the mean of the SMDs computed for each contrast weighted for sample size and the event rate. The Chi2 tests and the *I* ^2^ statistic were used to evaluate the degree of heterogeneity among the contrasts. The studies were considered remarkably heterogeneous if *I* ^2^ > 50%, and then a random effect model was used. Otherwise, a fixed effect model was employed. Additionally, subgroup analyses based on the timing of depression assessment, the type of stoke, and study location were performed to find the sources of heterogeneity. Meta‐regression analyses were applied to explore the causes of heterogeneity. Sensitivity analyses were conducted to assess the reliability of the results. Funnel plot and Egger's test were employed to evaluate potential publication bias.

## 3 RESULTS

### 3.1 Literature search and characteristics of eligible studies

**FIGURE 1:**
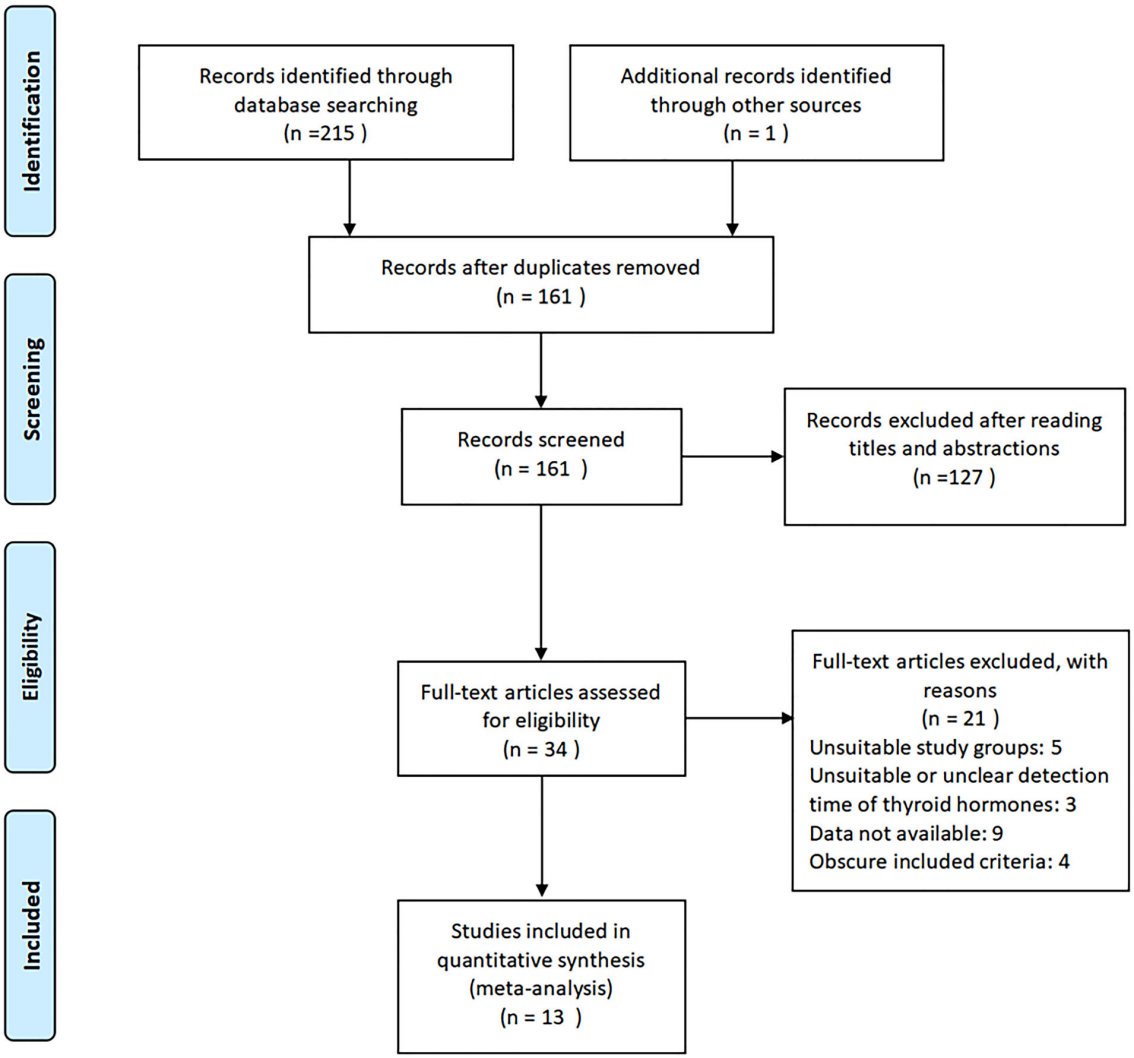
Search flow for the study identification and selection process.

The search flow diagram is shown in Figure 1. A total of 216 related literatures were obtained through the initial search. A total of 55 duplicates and 127 irrelevant studies were ruled out after the title and abstract screening, and 34 full‐text articles were identified for further scrutiny. Next, 21 studies were excluded due to inappropriate study groups, unclear detection time of thyroid hormones, unavailable data, or obscure inclusion criteria. Ultimately, 13 studies with 2122 subjects (736 in the PSD group and 1386 in non‐PSD group) were finally included in this meta‐analysis (Ahmed et al., 2020; Chen et al., 2008; Feng, 2021; Guo, Wang, Xia, et al., 2022; Li et al., 2005; Liu et al., 2022; Ma & Zhao, 2019; Taroza et al., 2019; Wang, 2009; Wang & Liu, 2011; Wei & Cui, 2016; Xia et al., 2020; Zhao, 2017). The publication times of included studies ranged from 2005 to 2022. Among the included studies, one was carried out in Saudi Arabia (Ahmed et al., 2020), one in Lithuania (Taroza et al., 2019), and the rest in China (Chen et al., 2008; Feng, 2021; Guo, Wang, Xia, et al., 2022; Li et al., 2005; Liu et al., 2022; Ma & Zhao, 2019; Wang, 2009; Wang & Liu, 2011; Wei & Cui, 2016; Xia et al., 2020; Zhao, 2017). TSH levels were detected in 12 of the studies, FT3 levels were assessed in 10 studies, and FT4 levels were tested in 9 studies. The total NOS scores ranged from 5 to 8 points in each study, reaching high‐quality standards. The characteristics of included studies are shown in Table 1.

**TABLE 1:** Characteristics of the included studies.

Study	Country	Age (mean, year)	Female	Type of stroke	Stroke assessment	Depression assessment	Time points of depression assessment	Number	Thyroid hormones (mean ± SD)	NOS score
		PSD non‐PSD	PSD non‐PSD					PSD non‐PSD	PSD non‐PSD	
Guo et al. (2022)	China	60.9 57.9	37.9% 29.5%	first‐ever AIS	MRI	DSM‐V (HAMD‐24)	1 week after stroke	87 285	FT3 (pmol/L): 4.34 ± 0.39 4.50 ± 0.46	7
									FT4 (pmol/L): 11.31 ± 2.91 11.15 ± 1.99	
									TSH (mlU/L): 2.28 ± 1.09 2.34 ± 1.61	
Liu et al. (2022)	China	64.14 65.01	30.3% 25.6%	AIS	MRI	HAMD ≥ 8	NA	66 117	TSH (μIU/mL): 2.19 ± 1.35 1.87 ± 0.83	6
Feng (2021)	China	57.22 56.47	46.7% 45.5%	first‐ever AIS	CT/MRI	PHQ‐9 (HDRS‐17 ≥ 7)	2 and 14 days after admission	45 55	FT3 (pg/mL): 1.18 ± 0.12 1.27 ± 0.12	7
									FT4 (ng/dL): 2.09 ± 0.18 1.81 ± 0.23	
									TSH (mlU/L): 0.15 ± 0.04 0.19 ± 0.04	
Xia et al. (2020)	China	66.4 63.1								
	48.4% 34.2%	first‐ever AIS	/	HAMD ≥ 8						
	3 months after discharge	128 117	FT3 (pmol/L): 4.009 ± 0.765 4.266 ± 0.700	8						
									FT4 (pmol/L): 16.782 ± 2.707 16.084 ± 2.957	
									TSH (mIU/L): 1.167 ± 0.479 3.003 ± 1.118	
Ahmed et al. (2020)	Saudi Arabia	57.22 56.44	50% 40.6%	AIS	/	HADS	3 months after discharge	18 32	TSH: 1.04 ± 1.23 3.28 ± 2.83	8
Ma and Zhao (2019)	China	57.85 57.21	36.2% 49.3%	AIS	Chinese guidelines	DSM‐IV (HAMD)	NA	58 148	TSH (μIU/mL): 1.05 ± 0.35 2.36 ± 0.96	6
Taroza et al. (2019)	Lithuania	69 68	45.5% 40%	AIS	CT/MRI	HADS‐D > 7	1 week after stroke	44 125	FT3 (pmol/L): 4.08 ± 0.64 4.37 ± 0.66	7
									FT4 (pmol/L): 16.63 ± 2.17 16.21 ± 2.51	
									TSH (mIU/L): 1.44 ± 1.33 1.64 ± 1.42	
Zhao. (2017)	China	62.51 62.21	46.3% 32.8%	Stroke	CT/MRI	HAMD‐17 > 7	1 month after stroke	95 259	FT3 (pmol/L): 3.89 ± 0.78 4.09 ± 0.77	7
									FT4 (pmol/L): 10.64 ± 2.17 9.99 ± 2.13	
									TSH (mIU/L): 1.43 ± 0.69 1.70 ± 0.89	
Wei and Cui (2016)	China	35.38 34.38	38.5% 44.3%	Stroke	CT/MRI	HAMD‐17 ≥ 7	NA	52 70	FT3 (ng/L): 2.52 ± 0.48 2.72 ± 0.60	6
Wang and Liu (2011)	China	61.5	45%	stroke	CT/MRI	HAMD‐17 ≥8	NA	28 32	FT3 (ng/mL): 1.28 ± 0.56 1.42 ± 0.64	5
									FT4 (pg/mL): 1.71 ± 0.62 1.65 ± 0.63	
									TSH (μIU/mL): 2.10 ± 0.68 2.73 ± 1.15	
Wang (2009)	China	—	—	Stroke	CT/MRI	HAMD‐17 > 16	3 weeks after stroke	45 45	FT3 (pmol/L): 3.64 ± 0.31 3.92 ± 0.44	7
									FT4 (pmol/L): 14.72 ± 1.77 15.24 ± 2.28	
									TSH (μIU/mL): 2.33 ± 0.87 2.18 ± 0.56	
Chen et al. (2008)	China	62.3	48.4%	AIS	CT/MRI	HAMD ≥ 8	NA	27 35	FT3 (ng/mL): 1.07 ± 0.37 1.28 ± 0.19	5
									FT4 (pg/mL): 1.83 ± 0.21 1.58 ± 0.24	
									TSH (μL/mL): 2.09 ± 0.36 2.27 ± 0.49	
Li et al. (2005)	China	60.2	37.6%	Stroke	CT/MRI	HAMD‐17 ≥ 8	NA	43 66	FT3 (ng/L): 1.32 ± 0.70 1.59 ± 0.84	5
									FT4 (ng/L): 1.77 ± 0.81 1.67 ± 0.64	
									TSH (mIU/L): 2.10 ± 0.68 2.50 ± 1.15	

Abbreviations: AIS, acute ischemic stroke; CT, computed tomography; DSM, diagnostic and statistical manual of mental disorders; FT3, free triiodothyronine; FT4, free thyroxine; HADS, hospital anxiety and depression scale; HAMD, Hamilton depression scale; HDRS, Hamilton depression rating scale; MRI, magnetic resonance imaging; NA, not available; NOS, Newcastle–Ottawa Scale; PHQ, patient health questionnaire; PSD, poststroke depression; SD, standard deviation; TSH, thyroid‐stimulating hormone.

### 3.2 Serum TSH levels and the risk of PSD

**FIGURE 2:**
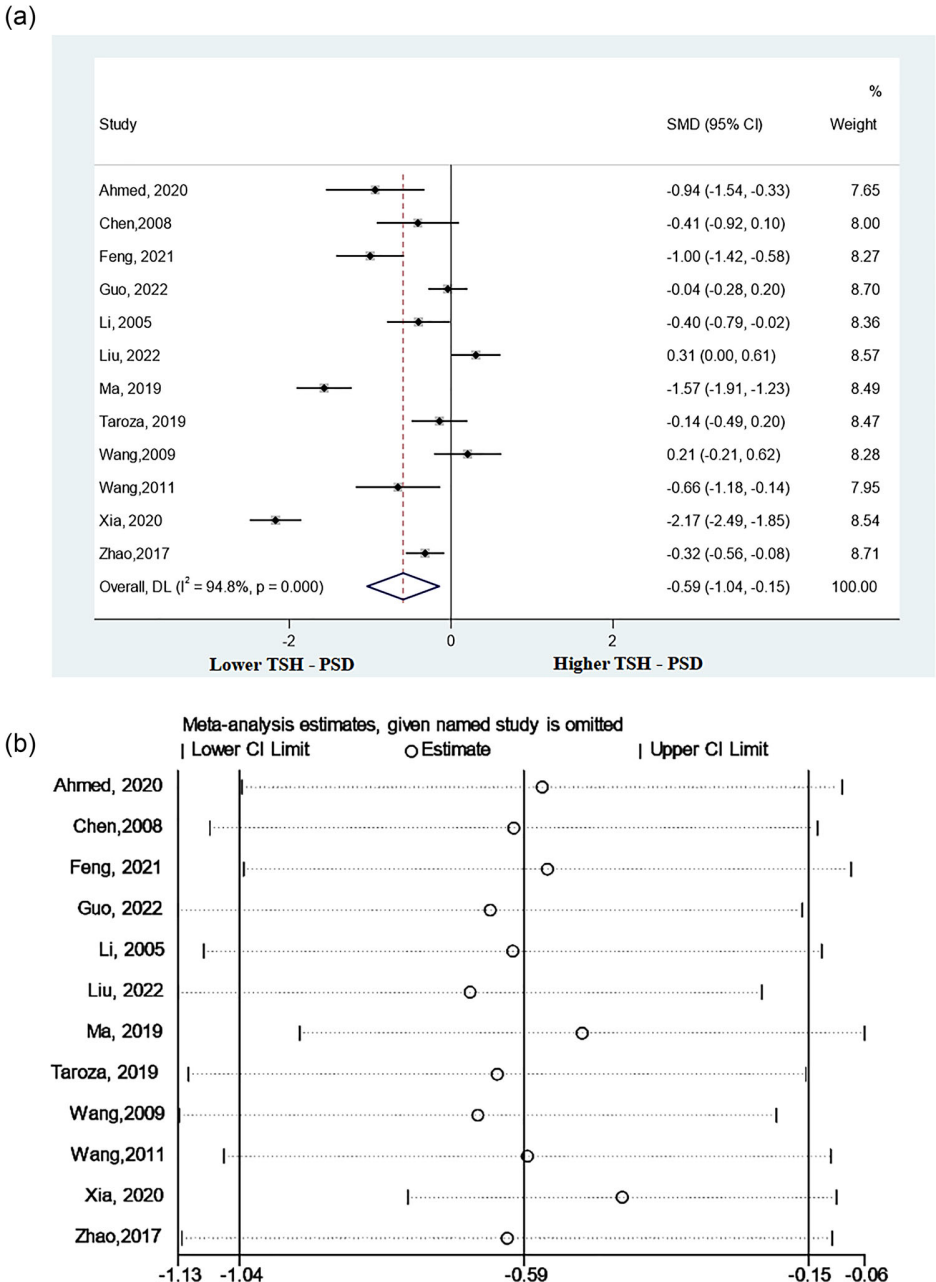
Results of meta‐analysis on thyroid‐stimulating hormone (TSH) levels in poststroke depression (PSD) patients and the non‐PSD: (a) forest plot indicated that the TSH levels were distinctly lower in PSD patients than in the non‐PSD and (b) sensitivity analysis indicated a reliable result of this meta‐analysis.

This meta‐analysis included 12 studies (Ahmed et al., 2020; Chen et al., 2008; Feng, 2021; Guo, Wang, Xia, et al., 2022; Li et al., 2005; Liu et al., 2022; Ma & Zhao, 2019; Taroza et al., 2019; Wang, 2009; Wang & Liu, 2011; Xia et al., 2020; Zhao, 2017) that detected serum TSH levels in patients with PSD versus non‐PSD patients. The results indicated that patients with PSD had lower levels of TSH compared to non‐PSD patients (SMD = −0.59, 95%CI = −1.04 to −.15, *p* = .009) (Figure 2). Additionally, significant heterogeneity was observed among the associated studies (*p* = .000, *I* ^2^ = 94.8%).

A sensitivity analysis was conducted to evaluate the effect of each study on total SMD. There was no significant change in combined SMD after removing any study, which indicated that the results of this analysis were reliable (Figure 2).

The results of the three subgroup analyses, based on the clinical differences of included studies, are demonstrated in Table 2. In the subgroup analysis according to the timing of depression assessment, five studies assessed the PSD within 1 month after stroke (≤1 month) (Feng, 2021; Guo, Wang, Xia, et al., 2022; Taroza et al., 2019; Wang, 2009; Zhao, 2017), and two studies assessed over 1 month after stroke (>1 month) (Ahmed et al., 2020; Xia et al., 2020). The time points for depression assessment in the other five studies were unclear (Chen et al., 2008; Li et al., 2005; Liu et al., 2022; Ma & Zhao, 2019; Wang & Liu, 2011). The TSH levels in patients of the PSD group were significantly lower than those of the non‐PSD group over 1 month after stroke (≤1 month: SMD = −0.25, CI 95% = −.57 to .07; >1 month: SMD = −1.58, CI 95% = −2.79 to −.38; unclear: SMD = −0.55, CI 95% = −1.26 to .17). In another subgroup analysis according to the type of stroke, PSD patients had significantly lower TSH levels compared to non‐PSD patients in the ischemic stroke subgroup (SMD = −0.74, 95% CI = −1.40 to −.09), which was not observed in the stroke group (SMD = −0.28, 95% CI = −0.58 to .02). Additionally, in the subgroup analysis according to study location, we found that the association between lower levels of TSH and the prevalence of PSD was more significant in China (SMD = −0.61, 95% CI = −1.12 to −.09).

**TABLE 2:** Results of subgroup analyses.

Index	Factors	No. of studies	SMD (95% CI)	*p* Value for heterogeneity	*I* ^2^ value
TSH					
	1. Time point of depression assessment				
	≤1 month after stroke	5	−.25 (−.57 to .07)	.000	80.5%
	>1 month after stroke	2	−1.58 (−2.79 to −.38)	.000	92.0%
	Unclear	5	−.55 (−1.26 to .17)	.000	93.9%
	2. Type of stroke				
	Ischemic stroke	8	−.74 (−1.40 to −.09)	.000	96.4%
	Stroke	4	−.28 (−.58 to .02)	.051	61.4%
	3. Study location				
	China	10	−.61 (−1.12 to −.09)	.000	95.6%
	Other countries	2	−.50 (−1.27 to .28)	.026	79.9%
FT3					
	1. Time point of depression assessment				
	≤1 month after stroke	5	−.42 (−.55 to −.28)	.155	40.0%
	>1 month after stroke	1	−.35 (−.60 to −.10)	–	–
	Unclear	4	−.40 (−.61 to −.18)	.527	0%
	2. Type of stroke				
	Ischemic stroke	5	−.44 (−.58 to −.30)	.349	10.0%
	Stroke	5	−.35 (−.51 to −.20)	.417	0%
	3. Study location China	9	−.40 (−.51 to −.29)	.337	11.7%
	Other countries	1	−.44 (−.79 to −.10)	–	–
FT4					
	1. Time point of depression assessment				
	≤1 month after stroke	5	.31 (−.09 to .71)	.000	87.6%
	>1 month after stroke	1	.25 (−.00 to .50)	–	–
	Unclear	3	.43 (−.17 to 1.02)	.009	79.0%
	2. Type of stroke				
	Ischemic stroke	5	.55 (.11–.98)	.000	88.2%
	Stroke	4	.15 (−.02 to .32)	.149	43.7%
	3. Study Location				
	China	8	.36 (.06–.65)	.000	83.3%
	Other countries	1	.17(−.17 to .52)	–	–

Abbreviations: CI, confidence intervals; FT3, free triiodothyronine; FT4, free thyroxine; No., numero; SMD, standard mean differences; TSH, thyroid‐stimulating hormone.

Meta‐regression analysis indicated that age and sex were not the causes of heterogeneity (Table 3).

**TABLE 3:** Results of meta‐regression.

Index	Variable	Coefficients	95%CI	*p*‐Value
TSH				
	Age in case group	.034	−.112 to .179	.613
	Age in control group	.066	−.074 to .206	.317
	Female proportion in case group	−.046	−.125 to .033	.223
	Female proportion in control group	−.039	−.103 to .025	.200
FT3				
	Age in case group	.001	−.015 to .016	.907
	Age in control group	.001	−.015 to .016	.933
	Female proportion in case group	−.006	−.035 to .024	.660
	Female proportion in control group	−.014	−.036 to .008	.168
FT4				
	Age in case group	−.058	−.173 to .058	.266
	Age in control group	−.048	−.175 to .078	.388
	Female proportion in case group	.053	−.041 to .148	.217
	Female proportion in control group	.046	−.006 to .099	.074

Abbreviations: CI, confidence intervals; FT3, free triiodothyronine; FT4, free thyroxine; TSH, thyroid‐stimulating hormone.

Funnel plot and Egger's test were employed to assess publication bias. As shown in Figure S1, the funnel plot of studies indicated the asymmetric distribution of scattered dots on both sides of the solid line, whereas the result of Egger's test was not significant (*p* > .05). There was no evidence of significant publication bias in the current meta‐analysis.

### 3.3 Serum FT3 levels and the risk of PSD

**FIGURE 3:**
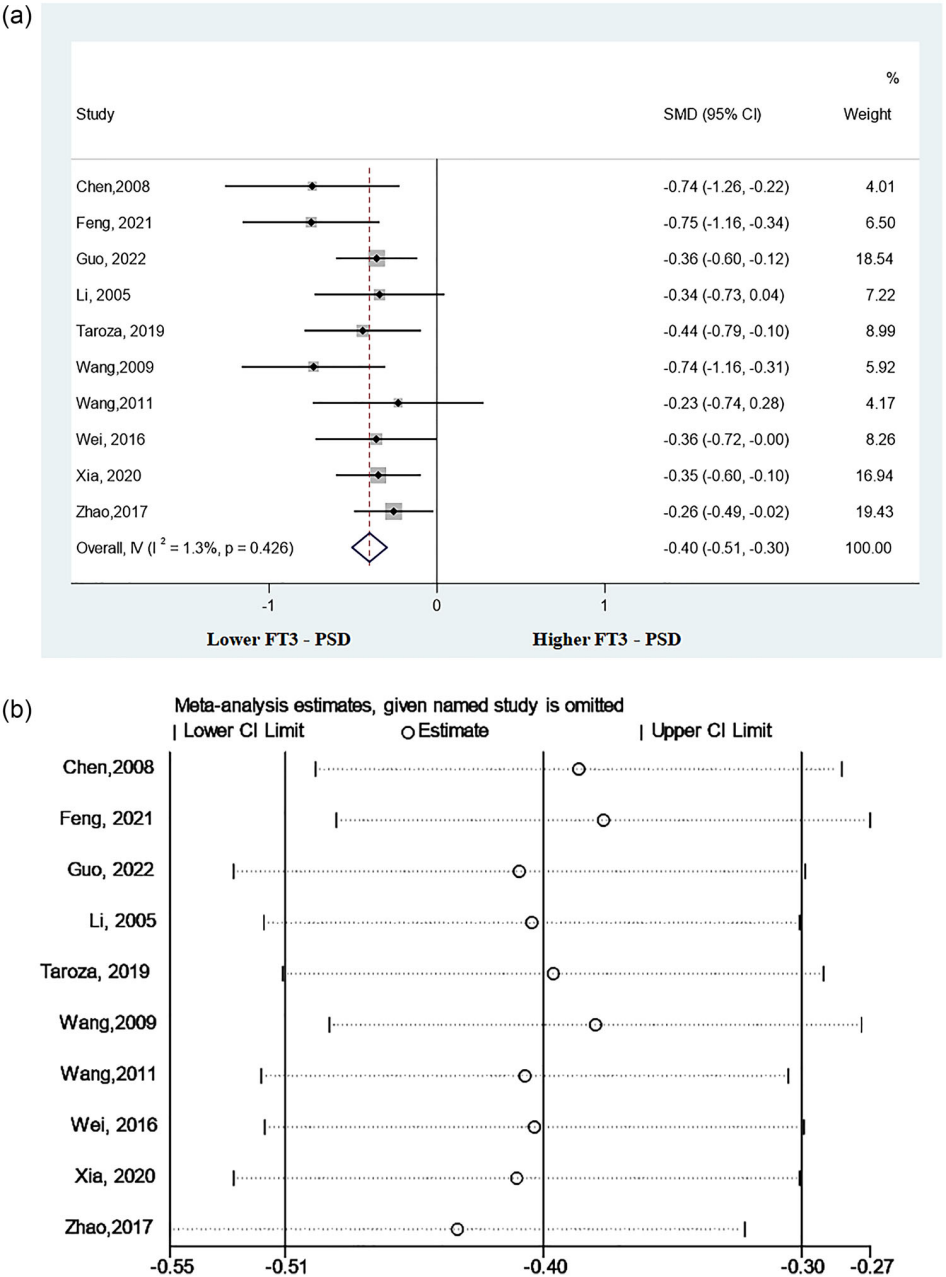
Results of meta‐analysis on free triiodothyronine (FT3) levels in poststroke depression (PSD) patients and the non‐PSD: (a) forest plot indicated that the FT3 levels were significantly lower in PSD patients than in the non‐PSD and (b) Sensitivity analysis indicated a reliable result of this meta‐analysis.

This meta‐analysis included 10 studies (Chen et al., 2008; Feng, 2021; Guo, Wang, Xia, et al., 2022; Li et al., 2005; Taroza et al., 2019; Wang, 2009; Wang & Liu, 2011; Wei & Cui, 2016; Xia et al., 2020; Zhao, 2017) that detected serum FT3 levels in patients with PSD versus non‐PSD patients. The results indicated that patients with PSD had lower levels of FT3 compared to non‐PSD patients (SMD = −0.40, 95%CI = −.51 to −.30, *p* = .000) (Figure 3). Additionally, no significant heterogeneity was observed among the associated studies (*p* = .426, *I* ^2^ = 1.3%).

A sensitivity analysis was performed to assess the effect of each study on total SMD. There was no remarkable change in combined SMD after removing any study, which indicated that the results of this analysis were stable (Figure 3).

The results of the three subgroup analyses are demonstrated in Table 2. All of the results suggested that serum FT3 levels in patients with PSD were significantly lower compared with non‐PSD patients regardless of the timing of depression assessment (≤1 month: SMD = −0.42, CI 95% = −.55 to −.28; >1 month: SMD = −0.35, CI 95% = −.60 to −.10; Unclear: SMD = −0.40, CI 95% = −.61 to −.18), type of stroke (ischemic stroke: SMD = −0.44, CI 95% = −.58 to −.30; stroke: SMD = −0.35, CI 95% = −.51 to −.20), or study location (China: SMD = −0.40, CI 95% = −.51 to −.29; other countries: SMD = −0.44, CI 95% = −.79 to −.10).

A meta‐regression analysis indicated that age and sex were not the sources of heterogeneity (Table 3).

As shown in Figure S2, the funnel plot of studies indicated the asymmetric distribution of scattered dots on both sides of the solid line, whereas the result of Egger's test was not significant (*p* > .05). Therefore, there was no potential risk of publication bias in the meta‐analysis.

### 3.4 Serum FT4 levels and the risk of PSD

**FIGURE 4:**
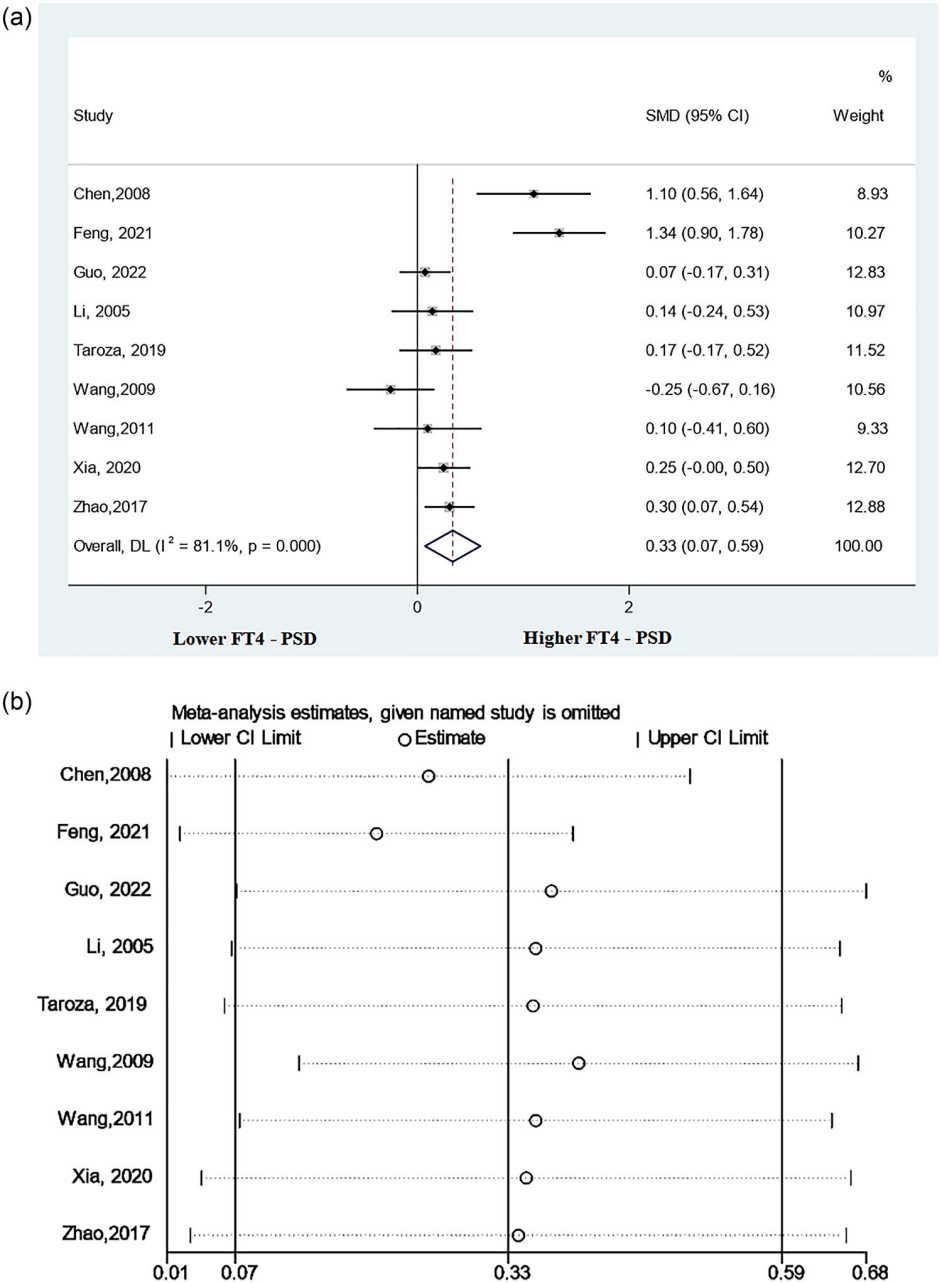
Results of meta‐analysis on free thyroxine (FT4) levels in poststroke depression (PSD) patients and the non‐PSD: (a) forest plot indicated that the FT4 levels were significantly higher in PSD patients than in the non‐PSD, and (b) sensitivity analysis indicated a reliable result of this meta‐analysis.

This meta‐analysis included nine studies (Chen et al., 2008; Feng, 2021; Guo, Wang, Xia, et al., 2022; Li et al., 2005; Taroza et al., 2019; Wang, 2009; Wang & Liu, 2011; Xia et al., 2020; Zhao, 2017) that detected serum FT4 levels in patients with PSD versus non‐PSD patients. The results indicated that patients with PSD had higher levels of FT4 compared to non‐PSD patients (SMD = 0.33, 95%CI = .07–.59, *p* = .013) (Figure 4). Additionally, significant heterogeneity was observed between the associated studies (*p* = .000, *I* ^2^ = 81.1%).

A sensitivity analysis was carried out to assess the effect of each study on total SMD. There was no significant change in pooled SMD after removing any study, which suggested that the results of this analysis were stable (Figure 4).

The results of the three subgroup analyses are indicated in Table 2. In the subgroup analysis according to the timing of depression assessment, all of the results indicated no remarkable difference in FT4 levels between patients of the PSD group and those of the non‐PSD group (≤1 month: SMD = 0.31, CI 95% = −.09 to .71; >1 month: SMD = 0.25, CI 95% = −.00 to .50; unclear: SMD = 0.43, CI 95% = −.17 to 1.02). In the subgroup analysis according to the type of stroke, PSD patients had significantly higher FT4 levels compared to non‐PSD patients in the ischemic stroke subgroup (SMD = 0.55, 95% CI = .11 to .98), which was not observed in the stroke group (SMD = 0.15, 95% CI = −.02 to .32). In another subgroup analysis according to study location, we found that the association between higher levels of FT4 and the prevalence of PSD was more significant in China (SMD = 0.36, 95% CI = .06–.65).

Meta‐regression analysis demonstrated that age and sex were not responsible for heterogeneity across studies (Table 3).

As shown in Figure S3, the funnel plot indicated the asymmetric distribution, whereas the result of Egger's test was not significant (*p* > .05). There was no significant risk of publication bias in this meta‐analysis.

## 4 DISCUSSION

To the best of our knowledge, our meta‐analysis for the first time explored the relationship and serum thyroid hormone levels in the acute phase of stroke and the risk of PSD. The current meta‐analysis indicated that, compared to patients without PSD, stroke patients with PSD had higher FT4 levels as well as lower TSH and FT3 levels. The results supported that serum thyroid hormones may become potential biomarkers for predicting the risk of PSD development in stroke survivors. The prevalence of PSD in the current study was 34.68%, which was similar to previous studies (Chen et al., 2022; Schöttke & Giabbiconi, 2015).

Thyroid dysfunction is associated with the occurrence of various neuropsychiatric disorders, including depression (Kafle et al., 2020). Both overt hypo‐ and hyperthyroidism are related to an increased risk of depression (Duntas & Maillis, 2013; Thomsen et al., 2005). In addition, some studies have also indicated the relationship among variations in thyroid hormone levels within the normal range and the risk of depression. An investigation by Medici et al. (2014) reported that the elderly with low‐normal TSH levels had an increased risk of developing depression. The possible mechanism of thyroid hormones associated with depression may be involved in the regulatory effects of thyroid hormones on serotonergic neurotransmission, which plays a critical role in depressive disorder (Bauer & Whybrow, 2021). Studies have indicated that exogenous thyroid hormones could increase cortical serotonin (5‐HT) concentrations and reduce the sensitivity of 5‐HT1A autoreceptors in the raphe area, leading to disinhibition of 5‐HT release, and thus exert modulatory effects on affective diseases (Bauer et al., 2002). Additionally, thyroid hormones may be involved in the pathophysiological process of depression by regulating the gamma aminobutyric acid (GABA) system. It is indicated that thyroid hormones influence multiple aspects of the GABA system, including the synthesis and degradation of GABA, the expression and function of GABA‐A receptor, as well as GABA release and reuptake (Karakatsoulis et al., 2021). Moreover, studies have demonstrated that the GABA system could participate in the pathogenesis of major depressive disorder by possible interactions with different processes involved in this disorder, including monoamine neurotransmission, hypothalamus–pituitary–adrenal axis, immune response, and neurotrophic factors (Bhandage et al., 2019; Della Vecchia et al., 2022; Slattery et al., 2005; Suzdak & Gianutsos, 1985). Thyroid abnormalities have been suggested to be associated with the occurrence of stroke and adverse functional outcomes (Squizzato et al., 2005; Wollenweber et al., 2013). It has been indicated that during the processes of brain reorganization after a stroke, mechanisms of brain development may be reactivated, which are associated with a series of cascades modulated by thyroid hormones (Talhada et al., 2019). Furthermore, thyroid status has an impact on neuronal death following stroke by regulating neuronal metabolic state and oxidative stress (Talhada et al., 2019), which could be the underlying mechanism of thyroid hormones affecting stroke outcomes. Therefore, no matter in depression or stroke, thyroid hormones seem to play an essential role, which may provide a theoretical basis for the relationship between thyroid hormone levels and the risk of PSD.

Of note, the levels of TSH, FT3, and FT4 revealed different performances in our subgroup analyses. Based on the results of the current subgroup analyses, we found a closer association and lower heterogeneity between lower FT3 levels and the risk of PSD, which suggested that serum FT3 could be a more stable biomarker for predicting PSD in stroke patients. One possible reason for this superiority may be that lower serum levels of FT3, as the unbound and biologically active form of thyroid hormone, have been reported to be negatively associated with neuroprotection for ischemia and the serotoninergic neurotransmitter system (Taroza et al., 2019), which could increase the risk of PSD development. Due to the limited number of included studies, our results should be considered with caution, which warrant additional research.

Apart from TSH, FT3, and FT4, thyroid autoantibodies are also commonly detected in the thyroid function test. Previous studies have indicated that thyroid autoantibodies are associated with both depression and stroke. van de Ven et al. (2012) reported that thyroid peroxidase antibodies were positively associated with trait markers of depression. Furthermore, a study from Shen et al. (2019) indicated that compared to non‐suicide attempters, suicide attempters had higher serum levels of anti‐thyroglobulin (TgAb) and thyroid peroxidases antibody (TPOAb) in first‐episode and drug‐naïve patients with major depressive disorder, and thus they proposed that TgAb and TPOAb could become promising biomarkers for predicting suicide risk in major depressive disorder. Additionally, elevated thyroid autoantibodies were reported to exacerbate stroke severity in euthyroidism with acute ischemic stroke, and the underlying mechanism may be involved in the vascular damage associated with thyroid autoimmunity, further increasing the risk of adverse outcomes (Li et al., 2022). In our included studies, only one study by Guo, Wang, Sun, et al. (2022) and Guo, Wang, Xia, et al. (2022) compared the levels of thyroid autoantibodies between PSD and non‐PSD patients, and they found that neither thyroid microsomal antibody nor TgAb was significantly different between the two groups. Therefore, the limited information does not permit us to conduct a meta‐analysis, and future studies can be implemented to further explore the association between the levels of thyroid autoantibodies and PSD.

The Chi2 test showed obvious heterogeneity in the meta‐analyses of serum TSH and FT4 levels and the risk of PSD. Subgroup analysis and meta‐regression analysis were applied to figure out the sources of heterogeneity. We observed that the current subgroup analyses based on the timing of depression assessment, the type of stoke, and study location did not completely clarify the high levels of heterogeneity. Additionally, previous studies have shown that gender and age were key factors for influencing the risk of PSD (Karaahmet et al., 2017; Zhang et al., 2017). Thus, meta‐regression analyses for age and sex were conducted, but the results indicated that age and sex were not the causes of heterogeneity in our meta‐analysis. Despite the obvious heterogeneity, the results of our meta‐analysis remain reliable, as each study included in our analysis reached a relatively high NOS score, and sensitivity analyses indicated no significant change in combined SMD after removing any study.

There are some limitations in the present meta‐analysis: (1) Some of the eligible studies in our meta‐analysis included patients with TIA, but strictly speaking, a TIA is not a true stroke, which causes temporary stroke‐like symptoms and fully resolves within 24 h. As these studies did not describe the specific number of patients with TIA, we could not conduct the relevant subgroup analysis to compare thyroid hormone levels of PSD patients with the non‐PSD in terms of different types of acute cerebrovascular diseases. (2) The definition of the acute phase of stroke varied from the first 3 days to the first month after stroke in these included studies, and some studies did not describe the specific period after acute stroke, which led to thyroid hormones being detected at different time points. However, due to the limited data, we did not analyze the differential relationships between thyroid hormone levels at different time points after acute stroke and the risk of PSD. (3) Great heterogeneity across studies was observed, which may influence the internal validity of meta‐analysis results. (4) Most of the included studies were conducted in China, and thus, extending our results to other nations should be considered with caution. (5) The follow‐up time was not so long in our included studies. Given that the prevalence of PSD remains high within a few years after stroke, studies with longer follow‐up time are needed in the future.

## 5 CONCLUSION

In summary, higher serum FT4 levels as well as lower serum TSH and FT3 levels in the acute phase of stroke may be associated with an increased risk of PSD in stroke patients without known thyroid diseases. Our study emphasizes the importance of thyroid screening tests in acute stroke survivors for the early identification of PSD.

### 5.1 Clinical implications

PSD is one of the frequent neuropsychiatric complications after stroke. The results of this meta‐analysis indicated that thyroid hormones, as the most frequently measured endocrine indexes, can probably predict PSD in stroke survivors. Patients with lower serum TSH and FT3 levels as well as higher serum FT4 levels in the acute stage of stroke may be more susceptible to PSD, and thus early and dynamic detection of PSD is necessary for such patients, in order to carry out early interventions. Additionally, future studies are needed to determine the efficacy of correction of thyroid abnormalities in acute stroke for the prevention of PSD.

## AUTHOR CONTRIBUTIONS

**Jie Fu**: Conceptualization; formal analysis; writing—original draft; methodology; data curation; investigation; software; funding acquisition. **Qin Zhao**: Data curation; investigation; methodology; formal analysis; validation. **Jinglun Li**: Data curation; investigation. **Xiu Chen**: Writing—review and editing. **Lilei Peng**: Conceptualization; supervision; writing—review and editing; funding acquisition.

## CONFLICT OF INTEREST STATEMENT

No conflict of interest to report.

### PEER REVIEW

The peer review history for this article is available at https://publons.com/publon/10.1002/brb3.3322.

## Supporting information


**Figure S1** The funnel plot (a) and Egger's test (b) for TSH and PSD. The funnel plot appeared to be asymmetric, but the result of Egger's test was not significant (*p* > .05), which suggested no publication bias in the current meta‐analysis.
**Figure S2** The funnel plot (a) and Egger's test (b) for FT3 and PSD. The funnel plot indicated the asymmetric distribution of scattered dots, whereas the result of Egger's test was not significant (*p* > .05). Therefore, there was no potential risk of publication bias in the meta‐analysis.
**Figure S3** The funnel plot (a) and Egger's test (b) for FT4 and PSD. The funnel plot indicated the asymmetric distribution, whereas the result of Egger's test was not significant (*p* > .05). There was no significant risk of publication bias in this meta‐analysis.

## Data Availability

The data used to support the findings of this study are included within the article.
