# Supplementary figures and images for: Association between thyroid hormone levels in the acute stage of stroke and risk of poststroke depression: A meta‐analysis
Source: Brain Behav. 2023 Dec 26;14(1):e3322. doi: 10.1002/brb3.3322 (PMC10751399; doi:10.1002/brb3.3322)

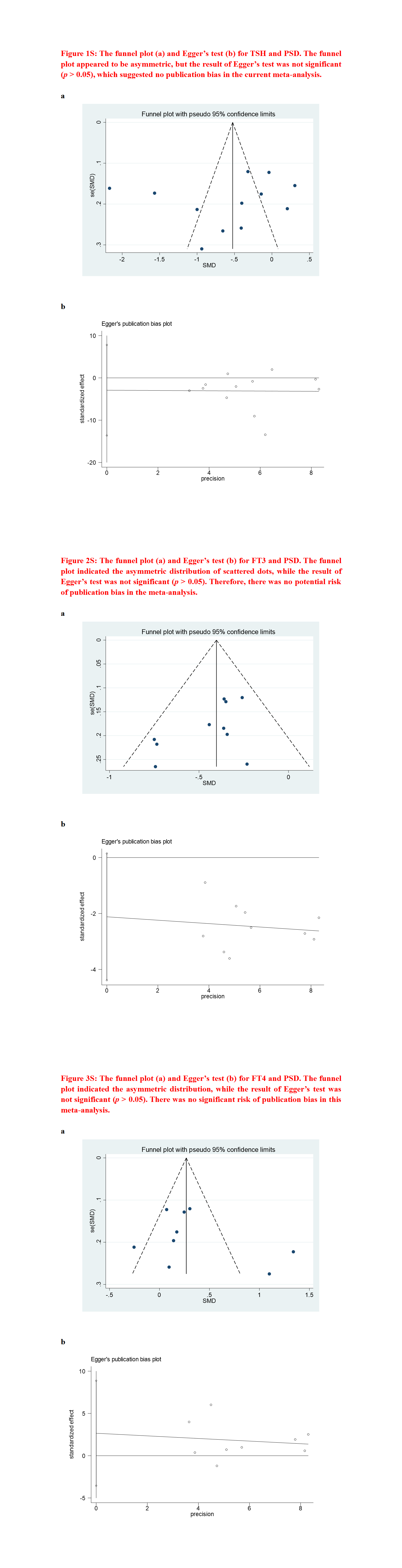

Supplement: Supplementary file 1 — Figure S1 The funnel plot (a) and Egger's test (b) for TSH and PSD. The funnel plot appeared to be asymmetric, but the result of Egger's test was not significant (p > .05), which suggested no publication bias in the current meta‐analysis. Figure S2 The funnel plot (a) and Egger's test (b) for FT3 and PSD. The funnel plot indicated the asymmetric distribution of scattered dots, whereas the result of Egger's test was not significant (p > .05). Therefore, there was no potential risk of publication bias in the meta‐analysis. Figure S3 The funnel plot (a) and Egger's test (b) for FT4 and PSD. The funnel plot indicated the asymmetric distribution, whereas the result of Egger's test was not significant (p > .05). There was no significant risk of publication bias in this meta‐analysis. [file BRB3-14-e3322-s001.tif]
